# Arabidopsis MORC proteins function in the efficient establishment of RNA directed DNA methylation

**DOI:** 10.1038/s41467-021-24553-3

**Published:** 2021-07-13

**Authors:** Yan Xue, Zhenhui Zhong, C. Jake Harris, Javier Gallego-Bartolomé, Ming Wang, Colette Picard, Xueshi Cao, Shan Hua, Ivy Kwok, Suhua Feng, Yasaman Jami-Alahmadi, Jihui Sha, Jason Gardiner, James Wohlschlegel, Steven E. Jacobsen

**Affiliations:** 1 Department of Molecular, Cell and Developmental Biology, University of California at Los Angeles, Los Angeles, CA USA; 2 Instituto de Biología Molecular y Celular de Plantas (IBMCP), CSIC‐Universidad Politécnica de Valencia, Valencia, Spain; 3 Eli & Edythe Broad Center of Regenerative Medicine & Stem Cell Research, University of California at Los Angeles, Los Angeles, CA USA; 4 Department of Biological Chemistry, UCLA, Los Angeles, CA USA; 5 Howard Hughes Medical Institute (HHMI), UCLA, Los Angeles, CA USA

**Keywords:** Chromatin, DNA methylation, Plant molecular biology

## Abstract

The Microrchidia (MORC) family of ATPases are required for transposable element (TE) silencing and heterochromatin condensation in plants and animals, and *C. elegans* MORC-1 has been shown to topologically entrap and condense DNA. In *Arabidopsis thaliana*, mutation of MORCs has been shown to reactivate silent methylated genes and transposons and to decondense heterochromatic chromocenters, despite only minor changes in the maintenance of DNA methylation. Here we provide the first evidence localizing Arabidopsis MORC proteins to specific regions of chromatin and find that MORC4 and MORC7 are closely co-localized with sites of RNA-directed DNA methylation (RdDM). We further show that MORC7, when tethered to DNA by an artificial zinc finger, can facilitate the establishment of RdDM. Finally, we show that MORCs are required for the efficient RdDM mediated establishment of DNA methylation and silencing of a newly integrated *FWA* transgene, even though *morc* mutations have no effect on the maintenance of preexisting methylation at the endogenous *FWA* gene. We propose that MORCs function as a molecular tether in RdDM complexes to reinforce RdDM activity for methylation establishment. These findings have implications for MORC protein function in a variety of other eukaryotic organisms.

## Abstract (web summary)

MORC ATPases are required for transposable element silencing and heterochromatin condensation in plants and animals. Here the authors show that Arabidopsis MORCs colocalize with sites of RNA-directed DNA methylation and provide evidence that they act as molecular tethers to efficiently establish DNA methylation.

## Introduction

Epigenetic chromatin modifications are fundamental to genome integrity, gene regulation, and transposon (TE) silencing. DNA methylation is a particularly stable epigenetic modification^1,2^, which in plants occurs in three sequence contexts, CG, CHG, and CHH (where H represents A, T, or C)^3^. Symmetric CG and CHG methylation are maintained by DNA METHYLTRANSFERASE1 (MET1) and CHROMOMETHYLASE3 (CMT3), respectively^4,5^, while asymmetric CHH methylation is mediated by CHROMOMETHYLASE 2 (CMT2) and DOMAINS REARRANGED METHYLTRANSFERASE1 and 2 (DRM1/2)^6^. The plant-specific RNA-directed DNA methylation (RdDM) pathway is required for both de novo and maintenance DNA methylation.

Canonical RdDM is composed of two major arms driven by either RNA Polymerase IV (Pol IV)^7^ or RNA Polymerase V (Pol V)^8^.

The Pol IV arm of the RdDM pathway features the generation of 24-nt siRNAs. Pol IV is recruited through its accessory proteins including the CLASSY SNF2-related putative chromatin remodelers (CLSYs) and the H3K9 methylation binding protein SAWADEE HOMEODOMAIN HOMOLOG 1 (SHH1)^9,10^. Pol IV generates transcripts that are converted into double-stranded RNAs (dsRNAs) by RNA-DEPENDENT RNA POLYMERASE 2 (RDR2)^11,12^. These dsRNAs are further cleaved by DICER-LIKE 3 (DCL3) into 24-nt siRNAs^13^, and are subsequently loaded onto the effector proteins ARGONAUTE 4 (AGO4), AGO6, or AGO9^14^.

The Pol V arm of the RdDM pathway constitutes the DNA methylation step. SUV(VAR)3-9 homologs SUVH2 and SUVH9 recognize preexisting DNA methylation and recruit the DDR complex^15–17^, consisting of DEFECTIVE IN MERISTEM SILENCING 3 (DMS3), DEFECTIVE IN RNA-DIRECTED DNA METHYLATION 1 (DRD1), and RNA-DIRECTED DNA METHYLATION 1 (RDM1)^18^. The DDR complex is required for the recruitment of Pol V, which generates non-coding scaffold RNAs that serve as a platform to recruit AGO4-siRNA effector complexes through sequence complementarity^8,18^. This effector complex in turn recruits DRM1/2 for de novo DNA methylation in all sequence contexts, leading to transcriptional gene silencing^6,19^.

Non-canonical RdDM pathways have also been described and are essential for the de novo methylation and silencing of newly integrated TEs and transgenes^20–23^. Pol II transcripts of TEs or other sequences can be processed by RDR6 and DCL2/4 to generate 21-22-nt siRNAs, which function primarily in post-transcriptional silencing but can occasionally trigger transcriptional silencing^20^ through the Pol V pathway, leading to the initial establishment of DNA methylation, after which canonical RdDM takes over to achieve full methylation and silencing of TEs.

Microrchidia (MORC) proteins are conserved GHKL (gyrase, HSP90, histidine kinase, MutL)-type ATPases involved in transcriptional gene silencing and chromatin compaction^24–32^. Mutations in MORC genes are associated with a number of human diseases, including Charcot-Marie-Tooth disease and cancer^30,33^. MORC proteins are required to repress TE expression in various organisms including human, mouse, *C. elegans*, and Arabidopsis^29,31,34^. In vitro studies recently showed that *C. elegans* MORC1 (ceMORC1) is able to topologically entrap and condense DNA (likely through encircling the DNA strands)^27^ and mutations of MORCs lead to visible chromatin decompaction in vivo in *C. elegans* and Arabidopsis^31,34^.

The *Arabidopsis thaliana* genome encodes six functionally redundant MORC proteins, MORC1, 2, 4, 5, 6, and 7^25^. MORC6 forms stable heterodimers with either MORC1 or MORC2^28^, whereas MORC4 and MORC7 are closely related and redundant proteins that form homodimers^25,34^. MORC5 is understudied because its expression is limited to pollen^32^. MORC6 mutations have been found in several independent forward genetic screens for factors involved in the maintenance of transcriptional gene silencing^24,34,35^. However, despite transcriptional upregulation of DNA methylated genes and transposons, Arabidopsis *morc* mutations show little effect on the maintenance of DNA methylation patterns. For instance, Moissiard et al. found no methylation changes in *morc* mutants either at the reactivated *SDC* transgene locus used for the mutant screen, or genome wide^34^. Lorkovic et al. found a moderate reduction of methylation in *morc6* at the 35S promoter in their screen^35^ and Brabbs et al. also reported moderate methylation reduction in the transgene used in their screen, but no methylation changes at some other *morc6* reactivated loci^24^. Similarly, Jing et al. identified *morc6* as a methylation independent repressor in their genetic screen^36^. A genome wide analysis of methylation patterns in a *morc* hextuple mutant (*morc1 morc2 morc4 morc5 morc6 morc7*, hereafter termed *hex*) eliminating all functional *MORC* genes came to a similar conclusion, as only a few hundred loci showed DNA methylation losses whereas the majority of the genome showed no changes in methylation despite transcriptional reactivation at many loci^25^. Similar but weaker gene expression changes and methylation losses were observed in *morc4 morc7* double mutants and *morc6* single mutants, indicating functional redundancy of the different MORC genes in Arabidopsis. Together these studies all suggest a role for MORCs in gene silencing, acting at least in part downstream of DNA methylation, but the relationship between MORCs and the DNA methylation pathways remains unclear.

Here we report the first genome-wide localization of MORCs in *A. thaliana* and find they are primarily localized to RdDM sites. Remarkably, MORC localization to these sites was stably maintained even in the absence of a functional RdDM machinery. However, it was lost in *met1* at sites that lost all DNA methylation, and redistributed to newly hypermethylated ectopic RdDM sites. We also found that ectopic recruitment of MORC7 to the *FWA* promoter was sufficient to cause de novo DNA methylation and transcriptional gene silencing, and this was dependent on functional RdDM. Surprisingly, despite no detectable loss of DNA methylation at the endogenous *FWA* locus in *morc* mutants, establishment of methylation on newly integrated *FWA* transgenes was greatly impaired in the absence of MORCs. This suggests that in addition to the role of MORCs acting downstream of DNA methylation, MORCs play a role in the efficient establishment of DNA methylation over newly integrated transgenes. We propose that MORCs act as chromatin tethers, loaded by the RdDM machinery, to facilitate the retention or re-recruitment of RdDM complexes at chromatin to promote efficient establishment of DNA methylation.

## Results

### MORC4 and MORC7 colocalize at RdDM sites

**Fig. 1:**
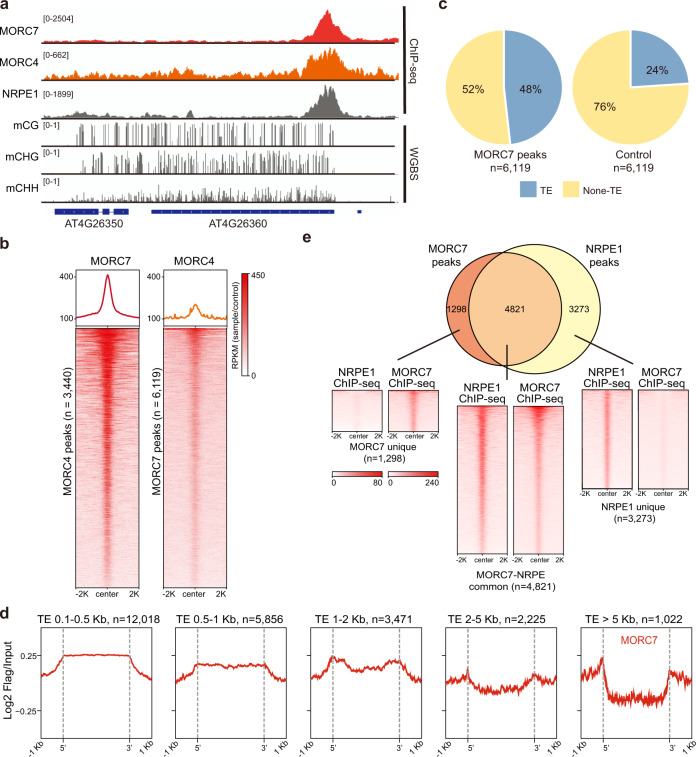
MORC4 and MORC7 largely co-localize at a subset of RdDM loci. **a** Screenshot of MORC4, MORC7, NRPE1 ChIP-seq (normalized by RPKM) and methylation level at a representative locus. **b** Metaplot and heatmap showing enrichment of MORC4 and MORC7 ChIP-seq signal over MORC7 (*n* = 6119) and MORC4 (*n* = 3440) peak regions, respectively. **c** Pie chart showing the percentage of MORC7 peaks overlapping with TEs. **d** Metaplot showing MORC7 ChIP-seq signal (log_2_ FLAG/input) over TEs categorized by different lengths. **e** Venn diagram (top) showing the relationship between MORC7 and NRPE1 peaks, and heatmap (bottom) showing the enrichment of MORC7 and NPRE1 over MORC7 unique (left, *n* = 1298), MORC7-NRPE1 common (middle, *n* = 4821) and NRPE1 unique (right, *n* = 3273) peaks.

To determine the genomic localization of MORC proteins, we performed chromatin immunoprecipitation (ChIP)-seq with pMORC4::MORC4-3xFLAG (MORC4-FLAG) and pMORC7::MORC7-3xFLAG (MORC7-FLAG) expressed in the their corresponding mutant backgrounds as reported previously^25,34^. MORC4 and MORC7 largely co-localized genome wide (Fig. 1a, b, Supplementary Fig. 1a). We identified 3440 and 6119 clear peaks for MORC4 and MORC7, respectively (MACS2, q value < 0.01, fold enrichment >2) with 80% of MORC4 peaks overlapping with MORC7 peaks (Fig. 1a, b, Supplementary Fig. 1b). The remaining 20% of the MORC4 “specific” peaks were also enriched in MORC7 (Supplementary Fig. 1b), but the level of enrichment did not pass our stringent peak calling threshold. MORC7 displayed stronger peak intensity at the majority of shared target regions. This is consistent with our previous report that MORC4 and MORC7 are close homologs that function largely redundantly to repress gene expression, with MORC7 playing a more dominant role^25^. Therefore, the absence of MORC4 ChIP signal over some MORC7 regions likely reflects the lower expression level of MORC4 and the sensitivity of ChIP. Given the stronger genetic effects as well as ChIP signal of MORC7, we focused on MORC7 for our subsequent analyses.

MORC7 was localized to regions that display strong characteristics of RdDM, including high levels of CHH methylation (Fig. 1a, Supplementary Fig. 1c, right panel) and enrichment over TEs, including the edges of long TEs and the entire length of short TEs (Fig. 1c, d). To further characterize the relationship with RdDM, we compared the binding patterns of MORC7 with that of the essential RdDM component, NRPE1^37^ and found that 80% of MORC7 peaks overlap with NRPE1 sites (Fig. 1e, Supplementary Fig. 1a). We defined these overlapping regions as MORC7-NRPE1 common peaks. Interestingly, about 20% of MORC7 peaks were devoid of NRPE1 and 40% of all NRPE1 peaks were devoid of MORC7. We define these two groups as MORC7-unique peaks and NRPE1-unique peaks, respectively (Fig. 1e, Supplementary Fig. 1d). MORC7-unique peaks are devoid of DNA methylation, more proximal to the transcriptional start sites of genes, and displayed higher levels of RNA Pol II signal compared to MORC7-NRPE1 common peaks (Supplementary Fig. 1c, e, f). The functional relevance of MORC7-unique peaks remains unclear.

To reveal the relationship between transcriptional repression and DNA methylation mediated by MORCs, we divided the genome into 100 bp bins, identified all bins that are upregulated in *hex*, merged continuous bins, and obtained 903 *hex* upregulated regions^25^. Among these 903 *hex* upregulated regions, 128 contain MORC7 binding sites within 1Kb (MORC7 bound), indicating that most of the upregulated regions in *hex* are not direct MORC targets (Supplementary Fig. 1g). Over these 128 MORC7 bound regions, we observed mild loss of DNA methylation in *hex* compared with *nrpe1* (Supplementary Fig. 1h). Interestingly, although loss of DNA methylation was much less in *hex* compared to *nrpe1*, transcriptional activation of these loci was much stronger in *hex* (Supplementary Fig. 1i). This result is consistent with the known role of MORCs in silencing downstream of DNA methylation^24,34,36^.

### MORC7 is associated with other MORCs and with RdDM components

To determine whether MORC7 associates with RdDM components, we performed immunoprecipitation followed by mass spectrometry (IP-MS) of MORC7-FLAG. Interestingly, we detected MORC4, MORC6, and MORC1 peptides, indicating that these MORC proteins interact in vivo (Table 1). This was in contrast to our previous report that MORC4 and MORC7 formed mutually exclusive homodimers and that MORC6 exclusively interacted with either MORC1 or MORC2^25,28^. This discrepancy likely reflects dramatic improvements in mass spectrometry sensitivity (Q-Executive instrument utilized in the previous study versus the Orbitrap Fusion Lumos used in the current study), and suggests that the interaction of MORC7 with MORC4, MORC6, and MORC1 may be weak or transient. In addition, we identified several RdDM components in the MORC7 IP-MS (Table 1). However, with the exception of DMS3, which was detected at higher abundance, relatively few peptides were detected for other RdDM components. This suggested that MORC7 interactions with RdDM components may be weak or transient. To test this, we adopted a crosslinked nuclear immunoprecipitation and mass spectrometry (CLNIP-MS)^38^ assay which stabilizes weak or transient interactions and captures proteins in close proximity in addition to directly interacting protein complexes. As a result, we observed a significant enrichment of both the MORCs and RdDM components (Table 1). Interestingly, without crosslinking, the MORC7 only pulled down components belonging to the Pol V arm of the RdDM pathway, and the CLNIP-MS consistently detected more peptides of Pol V arm components (Table 1). In addition, MORC6 has been reported to interact with DMS3 by in vitro co-immunoprecipitation^35^, and with SUVH9 and SWI3D by IP-MS and yeast two hybrid^36,39^. Together with our observation that MORC7 pulled down peptides of MORC1, 4, 6, and RdDM components in vivo, these data suggest that MORC proteins associate with the Pol V arm of the RdDM pathway, likely through the interaction with RdDM components, for instance, SUVH2/9 and DMS3.

**Table 1:** IP-MS of MORC7.

		Non crosslink (NSAFe5)				Crosslink (NSAFe5)			
ID	Protein	WT-1	WT-2	MORC7-1	MORC7-2	WT-1	WT-2	MORC7-1	MORC7-2
AT4G24970	MORC7	0	0	759	694	0	54	560	618
AT5G50780	MORC4	0	0	126	111	0	0	131	146
AT1G19100	MORC6	0	0	45	39	0	0	73	49
AT4G36290	MORC1	0	0	6	0	0	0	82	65
AT2G40030	NRPE1	0	0	3	2	30	10	81	114
AT3G49250	DMS3	5	4	43	41	0	0	91	77
AT4G13460	SUVH9	3	0	3	0	0	0	27	14
AT5G04290	SPT5L	0	0	2	1	0	0	74	74
AT3G48670	IDN2	0	0	6	6	0	0	102	78
AT1G21700	SWI3C	4	5	6	0	0	0	47	40
AT4G34430	SWI3D	0	0	3	5	0	0	39	37
AT5G14620	DRM2	0	0	0	0	0	0	11	0
AT2G16390	DRD1	0	0	0	0	0	0	27	26
AT2G33290	SUVH2	0	0	0	0	0	0	0	0
AT3G22680	RDM1	0	0	0	0	0	0	0	0
AT2G27040	AGO4	32	34	42	41	43	34	86	110
AT4G11130	RDR2	0	0	0	0	0	0	6	0
AT3G43920	DCL3	0	0	0	0	0	0	0	0
AT1G63020	NRPD1A	0	0	0	0	0	0	17	13
AT3G42670	CLSY1	0	0	0	0	0	0	0	0
AT1G15215	SHH1	0	0	0	0	0	0	0	0

Normalized spectral abundance factor value (NSAFe5) is indicated for each protein.

Source data are provided as IP-MS Source Data file.

Experiments were performed in two technical replicates.

### MORC7 can be recruited by the RdDM machinery

**Fig. 2:**
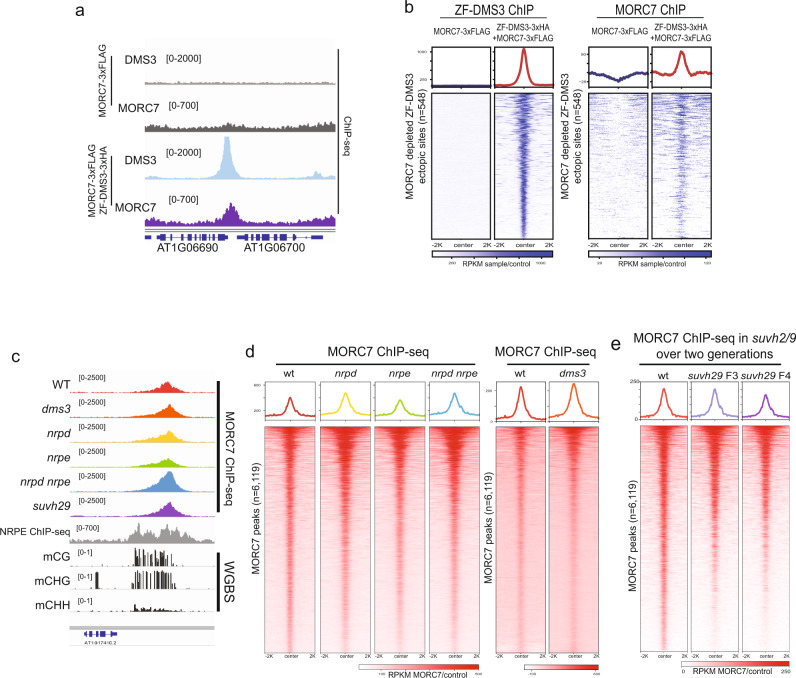
MORC7 can be recruited by RdDM but its maintenance is independent of RdDM machinery. **a** Screenshot of a representative ectopic ZF-DMS3 locus displaying enrichment of MORC7 (RPKM sample/control). **b** Metaplot and heatmap showing enrichment of MORC7 ChIP-seq signals (right panels) over a subset of ZF-DMS3 ectopic sites (left panels (RPKM sample/control) (*n* = 548). **c** Screenshot of MORC7 ChIP-seq (RPKM sample/control) in RdDM mutants (top six tracks), NRPE ChIP-seq (gray track), and methylation level (bottom three tracks) at a representative locus. **d** Metaplot and heatmap showing MORC7 ChIP-seq (RPKM sample/control) in RdDM mutants (*n* = 6119). **e** Metaplot and heatmap showing MORC7 ChIP-seq (RPKM sample/control) in *suvh2/9* over two generations (*n* = 6119).

Because MORC7 localized to RdDM sites and was associated with RdDM components, we next asked whether MORC7 can be recruited via the RdDM machinery. To test this, we utilized a fusion of DMS3 to an artificial zinc finger (ZF108) that was previously shown to target DMS3 to thousands of ectopic sites throughout the genome^40^. This leads to NRPE1 recruitment to these ectopic sites largely without triggering DNA methylation establishment^15,40^. Thus, if the RdDM machinery is sufficient to recruit MORC7, we expect MORC7 to also follow ZF108-DMS3 to these ectopic loci. We transformed UBQ10::ZF108-DMS3-3XHA into the MORC7-3XFLAG line and performed ChIP-seq for both ZF108-DMS3 and MORC7. We identified 548 ZF108-DMS3 ectopic sites with greater than fivefold enrichment of MORC7 in plants expressing ZF108-DMS3 but displaying no MORC7 signal in control plants (Fig. 2a, b), indicating that MORC7 can be efficiently recruited by the RdDM machinery to many ectopic sites in the genome.

### MORC7 maintenance at natural RdDM sites is independent of the RdDM machinery

Proper localization of RdDM components depends on the presence of a functional RdDM pathway. For example, Pol V association with chromatin requires both the DDR complex and SUVH2/SUVH9^41^, stability and localization of AGO4 are both disrupted in several RdDM mutants including *dcl3* and *rdr2*^42^, and Pol IV association with chromatin is reduced in the absence of SHH1^10^. Since MORC7 was found to be associated with RdDM proteins, we asked whether MORC7 displays the same characteristics as other RdDM components and whether its chromatin localization is abolished in the absence of RdDM machinery. To test this, we crossed the MORC7-FLAG line into several RdDM mutant backgrounds, including *dms3-4*, *nrpd1-4*, *nrpe1-11*, *nrpd1-4 nrpe1-11*, and *suvh2 suvh9*, and performed MORC7 ChIP-seq in each mutant background. Unexpectedly, MORC7 localization was virtually unperturbed in all these RdDM mutants backgrounds (Fig. 2c–e)^25^. We also analyzed the previously reported 519 RdDM sites that display partial loss of DNA methylation in *hex*^25^, and found that MORC7 retention on chromatin was largely unperturbed (Supplementary Fig. 2a, b). Thus, MORC7’s stable localization to chromatin does not require the major RdDM components including DMS3 which is part of the DDR complex, Pol V, Pol IV, or the SUVH2/9 proteins that are required for localization of the Pol V arm components of RdDM^15,41^.

### MORC7 retention on chromatin is dependent on DNA methylation or factor associated with DNA methylation

**Fig. 3:**
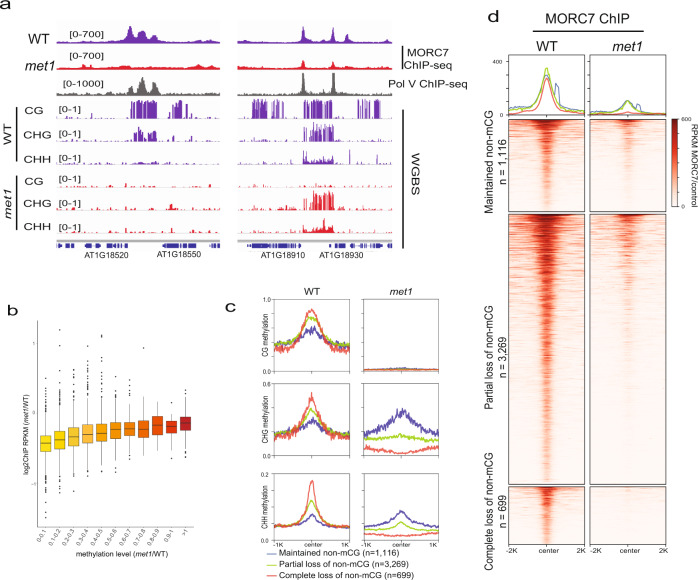
MORC7 maintenance is dependent on DNA methylation. **a** Screenshots of MORC7 ChIP-seq (RPKM MORC7/control) in WT (first track) and *met1* (second track) over two representative loci representing complete (left) and partial (right) loss of DNA methylation in *met1*. **b** Box plot showing MORC7 maintenance level (log2 MORC7 RPKM *met1*/WT) as categorized by total methylation level in *met1* (total methylation *met1*/WT). Center line indicates the median, upper and lower bounds represent the 75th and the 25th percentile, respectively, whiskers indicate the minimum and the maximum, and outliers are represented as circles. **c** Profile of CG (top panel), CHG (middle panel), and CHH (lower panel) methylation over MORC7 ChIP-seq peaks categorized by the level of non-CG methylation loss in *met1*. **d** Profile and heat map of MORC7 ChIP-seq signal (RPKM MORC7/control) in WT (left) and *met1* (right) over the three categories defined in **c**.

Although CHH methylation is eliminated in RdDM mutants at RdDM/MORC7 binding sites, CG and CHG methylation are largely retained^43^. We speculated that this remaining methylation, or another chromatin characteristic associated with methylation, might be sufficient to maintain MORC7. To test this, we performed MORC7 ChIP-seq in *met1* which shows complete elimination of CG methylation genome-wide. Due to inter-dependencies of DNA methylation pathways^2^, complete loss of DNA methylation is also observed over many regions^43^. We observed a dramatic reduction of MORC7 ChIP signal throughout the genome in *met1* (Fig. 3a). We examined the genome-wide correlation between changes in MORC7 occupancy and DNA methylation in *met1* (*met1*/WT) and observed a clear positive correlation, indicating that regions that retained more DNA methylation in *met1* also retained more MORC7 (Fig. 3b). Importantly, regions that lost DNA methylation completely in all sequence contexts in *met1* also lost all MORC7 binding (Fig. 3c, d). Together, these data indicate that MORC7 localization depends on DNA methylation or a factor associated with DNA methylation. MORC7 is unlikely to be recruited by DNA methylation directly because it is not present at other methylated sites, for instance at gene bodies, that are only methylated at CG sites. Instead it seems more likely that histone marks or variants associated with heterochromatin might be involved in the retention of MORC7.

### MORC7 is recruited to ectopic RdDM sites in *met1*

**Fig. 4:**
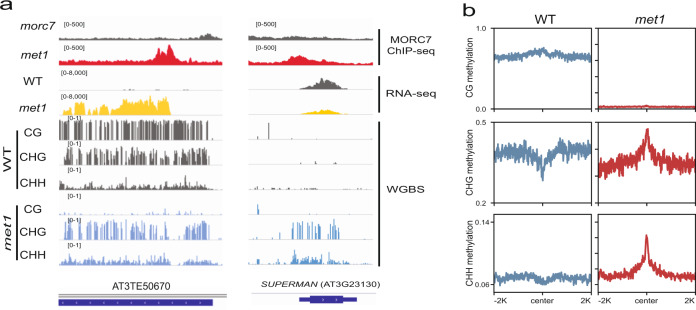
MORC7 is recruited to ecotpic RdDM sites in met1. **a** Screenshot of one locus that ectopically gained MORC7 in *met1* over a reactivated TE (left) and another example of ectopically gained MORC7 in *met1* over the *SUPERMAN* locus. Expression level of the corresponding TE in wild type (gray) and *met1* (yellow) were included. Methylation level in the wild type and *met1* were indicated in the bottom 6 tracks. **b** Methylation level over the ectopically gained MORC7 peaks in wild type (left) and *met1* (right).

Interestingly, we observed ectopic gain of MORC7 over certain regions in *met1*. Some of these sites corresponded to reactivated TEs (Fig. 4a). Expression of TEs may trigger non-canonical RdDM through the Pol II-RDR6 pathway, leading to the synthesis of siRNAs and the establishment of RdDM^22,44,45^. We observed 903 ectopically gained MORC7 peaks in *met1*, and found that these peaks were also associated with hyper-methylation in both CHG and CHH sequence contexts in *met1* (Fig. 4b), suggesting that these ectopic MORC7 peaks are likely a consequence of ectopic RdDM activity. For example, *SUPERMAN* is a well-characterized non-TE locus that becomes targeted by RdDM ectopically in *met1*^46^. As expected, we observed an ectopic gain of MORC7 over the promoter of *SUPERMAN* along with non-CG hyper-methylation in *met1* (Fig. 4a)^47^. Collectively, these observations further support our conclusion that MORC7 can be recruited by the RdDM machinery.

### ZF108-MORC7 represses *FWA* expression through RdDM

**Fig. 5:**
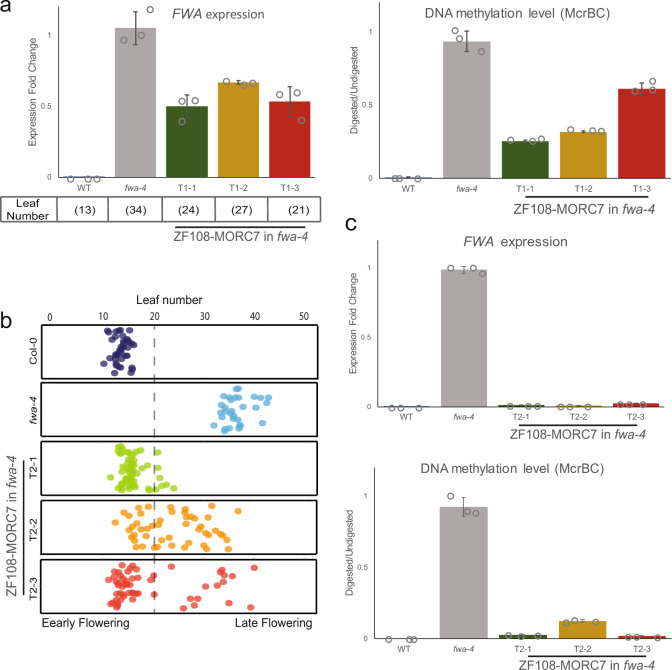
ZF108-MORC7 silences FWA expression through RdDM. **a** qRT-PCR analysis showing *FWA* expression level relative to *fwa-4* (left panel) in three independent ZF108-MORC7 T1 plants that displayed early flowering phenotype. Leaf numbers of these plants are indicated below the bar graph. McrBC analysis with the *FWA* promoter of the same plants is shown in the right panel. Error bars represent the mean ± s.d. of 3 technical replicates. **b** Flowering time of Col-0, *fwa-4*, and T2 progenies of the three early flowering T1 plants shown in **a**. **c** qRT-PCR of *FWA* expression level relative to WT (top) and McrBC analysis of the *FWA* promoter (bottom) of one representative plant in each T2 line displayed in **b**. Error bars represent the mean ± s.d. of 3 technical replicates. Source data underlying Fig. 5 are provided as a Source Data file.

*FWA* is normally methylated and silent^48,49^. However, the *fwa-4* epiallele that has stably lost DNA methylation is highly transcribed, leading to a heritable late flowering phenotype. We previously found that targeting MORC6 or MORC1 to *FWA* in an *fwa-4* background using ZF108 was able to trigger RdDM-dependent de novo DNA methylation and silencing of *FWA*^40^. To test whether MORC7 can also silence *FWA*, we fused ZF108 to MORC7 and transformed the fusion protein into *fwa-4*. Indeed, we observed an early flowering phenotype in T1 plants carrying UBQ10::ZF108-MORC7 indicating transcriptional silencing of *FWA*. We examined three early flowering T1 plants and confirmed partial repression of *FWA* by RT-qPCR and an increase in DNA methylation over the *FWA* promoter (Fig. 5a), as well as ectopic establishment of DNA methylation over some of the ZF ectopic sites (Supplementary Fig. 3a). We also followed these three early flowering T1 plants to the next (T2) generation and observed even earlier flowering (Fig. 5b), stronger repression of *FWA*, and a higher level of DNA methylation over the *FWA* promoter (Fig. 5c).

To test whether MORC7 is able to repress gene expression in the absence of RdDM, we transformed ZF108-MORC7 into *nrpe1* introgressed into the *fwa-1* background (*fwa-1 nrpe1-1*)^40^. We did not observe early flowering plants in the T1 generation and failed to detect significant repression of *FWA* in five independent ZF108-MORC7 expressing T1 lines (Supplementary Fig. 3b, c). Furthermore, we followed three T1 lines to the T2 generation and confirmed that *FWA* expression was not repressed (Supplementary Fig. 3d). These results show that MORC7-mediated repression of *FWA* expression is dependent on RdDM, which is similar to the reported behavior of ZF108-MORC6^40^.

### Efficient transgene silencing is impaired in *morc* mutants

Because MORCs are able to repress *FWA* gene expression through the recruitment of RdDM, and RdDM is responsible for the establishment of DNA methylation^50^, we asked whether MORCs may be required for the establishment of DNA methylation. To test this, we transformed plants with an *FWA* transgene, which in wild type is immediately and efficiently targeted for de novo DNA methylation and transcriptional silencing, leading to an early flowering phenotype^50^. However, in strong RdDM mutants, like *nrpe1*, de novo silencing of the *FWA* transgene is abolished^16,50,51^ and plants remain late flowering due to *FWA* overexpression. Considering the known functional redundancy of MORC proteins^25^, we transformed the *FWA* transgene into the *hex* mutant in which all functional *MORC* genes are mutated^25^.

**Fig. 6:**
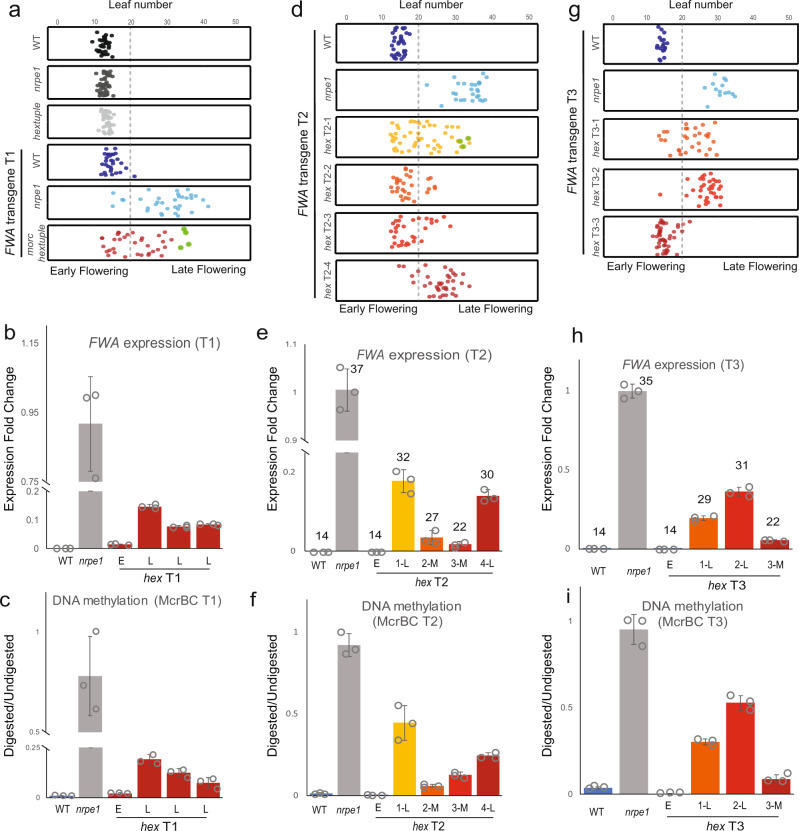
Transgene silencing is impaired in morc mutants. **a** Flowering time of T1 plants in wild-type, *nrpe1-11*, and *morc hex* backgrounds as indicated by the leaf number. The green dots represent the four late flowering plants propagated to the T2 generation. **b** qRT-PCR of *FWA* expression of four independent T1 plants in *hex* backgournd (crimson) relative to that in *nrpe1* background (gray). Error bars represent the mean ± s.d. of 3 technical replicates. **c** McrBC analysis of the *FWA* promoter of the same representative T1 plants displayed in **b**. Error bars represent the mean ± s.d. of 3 technical replicates. E represents early flowering and L represents late flowering. **d** Flowering time of T2 plants in wild-type, *nrpe1-11*, and the progenitor lines of the four late flowering T1 *hex* plants as indicated by the leaf number. The green dots in line T2-1 represent three late flowering T2 plants propagated to their T3 generation. **e** qRT-PCR of *FWA* expression level of one representative early and three late flowering plants in T2 progenitor lines in *hex* background, relative to that in *nrpe1* background. Leaf number of the corresponding plant is indicated. E early flowering, M intermediate flowering, L late flowering. Error bars represent the mean ± s.d. of 3 technical replicates. **f** McrBC analysis of the *FWA* promoter of the same T2 progenitor lines displayed in **e**. Error bars represent the mean ± s.d. of 3 technical replicates. E represents early flowering and M indicates intermediate flowering. E early flowering, M intermediate flowering, L late flowering. **g** Flowering time of T3 plants in wild-type, *nrpe1-11*, and the progenitor lines of the three late flowering T2-1 *morc hex* line as indicated by the leaf number. E early flowering, M intermediate flowering, L late flowering. **h** qRT-PCR of *FWA* expression level of one early flowering and three representative late flowering plants in T3 progenitors derived from T2-1. Leaf number of the corresponding plant is indicated. Error bars represent the mean ± s.d. of 3 technical replicates. E early flowering, M intermediate flowering, L late flowering. **i** McrBC analysis of the *FWA* promoter of the same representative T3 plants displayed in **i**. Error bars represent the mean ± s.d. of 3 technical replicates. E early flowering, M intermediate flowering, L late flowering. Source data underlying Fig. 6 are provided as a Source Data file.

In the T1 generation, we observed 59% (22/37) late flowering plants in *hex*, compared to 88% (29/33) and 3% (1/32) in the *nrpe1* and wild-type controls, respectively (Fig. 6a). As expected, late flowering phenotype was associated with *FWA* transgene overexpression as well as reduced DNA methylation establishment over the *FWA* promoter (Fig. 6b, c). This indicates that the efficiency of *FWA* transgene silencing through RdDM is greatly reduced in *hex*.

To determine if *FWA* transgene silencing might be established in subsequent generations, we followed four late flowering T1 *hex* plants to the T2 generation. Two out of the four T2 lines converted to mostly early/medium flowering (T2-2, T2-3) while the other two remained medium/late flowering (T2-1, T2-4) (Fig. 6d). We chose one late flowering plant from each T2 line and one early flowering plant from T2-2 and observed a clear association between the flowering time and the expression and methylation status, with late flowering plants displaying lower levels of DNA methylation and higher levels of *FWA* expression (Fig. 6e, f). Therefore, although efficient establishment of transgene silencing is strongly impaired in the *hex*, stochastic intergenerational silencing still occurs within the population.

We also asked whether the stochastic silencing of some lines but not others might be due to the location of the transgene within the genome. To test this, we chose three late flowering plants from the *hex* T2-1 population, which carries the *FWA* transgene at a single insertion site as indicated by a 3:1 segregation (hygromycin resistant:sensitive ratio in the T2 was 67:21) and followed these plants to the T3 generation. We observed early flowering in one T3 line (T3-3), while the other two (T3-1 and T3-2) remained mostly late flowering (Fig. 6g). Since all three have the same insertion, the stochastic silencing is likely a property of the *hex* mutant background, rather than a reflection of the transgene location within the genome. We also performed methylation and expression analysis on one early and three late flowering plants from each of the T3 lines, and consistently found that late flowering plants displayed a lower level of DNA methylation and higher level of *FWA* expression (Fig. 6h, i). These results show that *FWA* methylation and silencing failed to establish efficiently in *hex* in the T3 generation. In contrast to *hex*, the strong RdDM mutant, *nrpe1*, showed late flowering T1 plants that remained late throughout all generations tested (Fig. 6a, d, g), displaying no stochastic silencing.

To examine the establishment of DNA methylation specifically over the transgene in more detail, we performed bisulfite PCR (BS PCR) over two regions of the *FWA* promoter with the same T2 and T3 plants used for McrBC. The *FWA* transgene used for these experiments carries single nucleotide polymorphisms which allowed us to distinguish endogenous- and transgene-derived reads. In the CG context, most transgene-derived reads were fully unmethylated in *nrpe1*, while few fully unmethylated reads were detected in wild type (Supplementary Fig. 4a), demonstrating that the transgene was efficiently silenced in wild type but not in *nrpe1* as expected. *hex* plants displayed intermediate levels of unmethylated transgene-derived reads, with late flowering *hex* plants having more fully CG-unmethylated reads than early flowering *hex* in both T2 and T3 (Supplementary Fig. 4a, upper panels). We also detected more fully CHH-unmethylated reads in all *hex* lines over the transgene compared to the endogenous locus, consistent with *hex* inhibiting RdDM (Supplementary Fig. 4a lower panels, 4b). We did not analyze CHG methylation given the paucity of CHG sites at *FWA*. We also found that maintenance of preexisting DNA methylation over the endogenous *FWA* promoter was completely unaffected in *hex*, whereas RdDM mutants such as *nrpe1* and *suvh2 suvh9* lost most non-CG methylation as expected (Supplementary Figs. 4 and 5). Thus MORCs are required for efficient RdDM-mediated establishment of DNA methylation of newly integrated *FWA* transgenes, even though maintenance of preexisting DNA methylation at the same *FWA* sequences at the endogenous gene is completely unaffected in the *hex* mutant.

## Discussion

In this study we show that Arabidopsis MORC7 is localized to sites of RdDM, and likely associates with the Pol V arm of the RdDM pathway. Tethering of MORC7 to the unmethylated *FWA* locus with an artificial zinc finger was sufficient to recruit RdDM, methylation, and silencing to *FWA*. Conversely, tethering DMS3 to ectopic sites in the genome using an artificial ZF was sufficient to recruit MORC7. Furthermore, MORC7 was recruited to ectopic RdDM sites in *met1*. Together, these data suggest a mutual interaction and recruitment between MORC7 and RdDM. Finally, we found that a *morc* hextuple mutant is defective in the establishment of RdDM-mediated DNA methylation and silencing of a newly introduced *FWA* transgene, suggesting that MORCs facilitate the efficiency of RdDM activity during this process.

The MORC gene family in Arabidopsis consists of six potentially active genes, and several pieces of evidence suggest that the other MORCs have similar properties as we have observed here for MORC7. First, MORC4 was found to co-localize with MORC7 over RdDM sites. Second, our IP-MS and crosslink IP-MS show interactions between MORC7 and MORC4, MORC1 and MORC6, suggesting they likely function together. Third, consistent with our IP-MS data showing MORC7 association with Pol V arm RdDM components, MORC6 was previously shown to interact with two Pol V arm components, DMS3 and SUVH9^35,39^. Fourth, similar to ZF108-MORC7, ZF108-MORC1 and ZF108-MORC6 were previously shown to recruit RdDM to the *FWA* locus^40^, consistent with an interaction between MORC complex and the RdDM machinery. Finally, comparison of *hex* to *morc4 morc7* and *morc6* showed genetic redundancy between these family members^25^.

Given their association with RdDM components and their localization to RdDM sites, the MORCs are clearly acting in some capacity as RdDM factors. However, relative to known RdDM factors, the MORCs have unique genetic properties, in that they show strongly impaired efficiency of DNA methylation and silencing of transgenes, yet show very little effect on the maintenance of preexisting methylation at RdDM sites. All RdDM mutants that have been studied to date, including very weak mutants such as *frg1 frg2*, *suvr2*, and *drm3* show significant losses of methylation at the majority of RdDM sites^52^. However, an analysis of the *morc hex* mutant showed only a very small number of sites with any loss of methylation. Notably, although the *morc hextuple* mutant shows greatly impaired DNA methylation establishment of *FWA* sequences when new transgenes are introduced into plants, the *morc hextuple* has no defect in the maintenance of preexisting methylation of similar sequences at the endogenous *FWA* gene, while all other known RdDM mutants do (Supplementary Fig. 5). Another interesting difference is that while other RdDM components studied dissociate from chromatin in the absence of a functional RdDM machinery (e.g., Pol V dissociates from chromatin in the absence of the DDR complex, Pol IV is lost in the absence of SHH1, and AGO4 is lost in the absence of DCL3 or RDR2), MORC7 maintenance on chromatin was not disrupted in RdDM mutants.

**Fig. 7:**
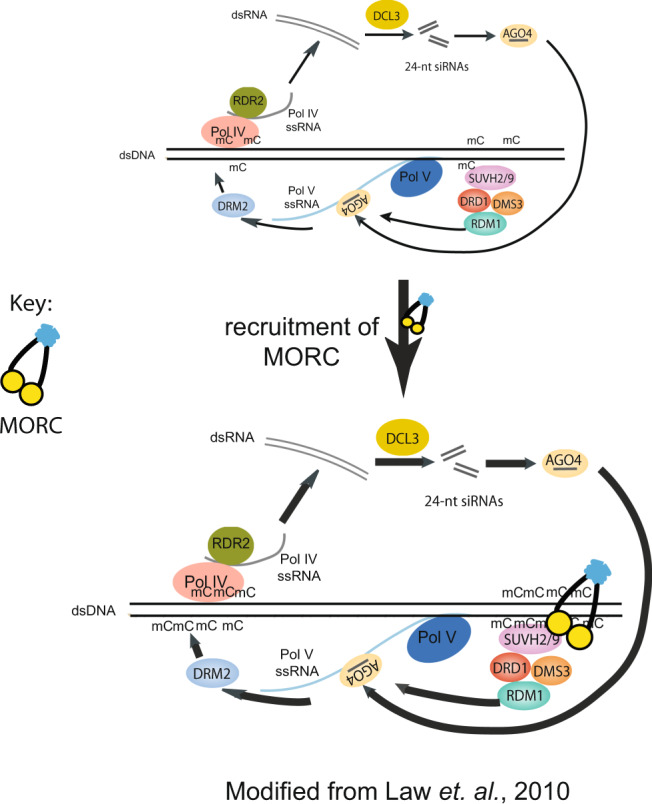
Working model of MORC proteins. A working model of MORC proteins in plant: MORC proteins are localized to RdDM sites through direct interaction with RdDM components. Upon recruitment, MORCs topologically entrap chromatin through dimerization and clamp around DNA to continuously reinforce the recruitment of RdDM complexes to their site of action and thereby, facilitate the efficiency and activity of the RdDM machinery.

To account for these unique properties, we suggest a model (Fig. 7a) in which MORCs are loaded onto sites of RdDM via interaction with RdDM components, after which they stay stably associated with these sites. One possible mechanism is that the Arabidopsis MORC proteins may be able to topologically entrap the chromatin at these sites by clamping around the DNA, similar to what has been shown for *C. elegans* MORC-1^27^. In this way, MORCs may serve as “tethers”, or a memory component, to facilitate the continuous recruitment of RdDM complexes back to sites after their initial targeting by RdDM. This tethering process may be mainly important during the early stages of the establishment of RdDM, which would explain why MORCs are required for efficient establishment of methylation at *FWA*, but not for maintenance of preexisting methylation at these same sequences. After initial establishment of methylation, and in the presence of strong maintenance of DNA methylation activity, MORCs may no longer be required for continued RdDM recruitment because of factors such SUVH2 and SUVH9 which bind to methylated DNA to continually recruit Pol V, and SHH1 which binds to H3K9 methylation to recruit Pol IV^9,10,15^.

An RdDM tethering model could also explain the effects of *morc* mutants on maintenance DNA methylation at a small subset of sites. A previous analysis of the *morc hextuple* mutant identified 519 sites, representing around 5% of RdDM sites, showing losses of methylation^25^. These “fragile sites” display a loss of DNA methylation as well as transcriptional activation in the absence of MORCs, as well as in RdDM mutants^25^. These fragile sites were shown to possess lower levels of symmetrical CG and CHG sequences compared to the genome average and thus are likely to show weakened CG and CHG maintenance DNA methylation^25^. We suggest that these sites may be fragile to the loss of MORCs because of transcriptional reactivation in the *morc* mutants, leading to a need for continued recruitment of RdDM to these sites in the face of weaker than average reinforcing maintenance of DNA methylation at symmetrical sites. MORC tethering to these sites may provide the needed boost in RdDM recruitment to maintain methylation and silencing.

Our findings on the biology of the Arabidopsis MORCs could have implications for the study of MORCs in other organisms. For instance mouse MORC1 is required for de novo DNA methylation and silencing of evolutionarily young and expressed TEs that are more recalcitrant to de novo methylation over the course of epigenetic reprogramming in primordial germ cells^29^. A MORC tethering model could nicely explain the continued recruitment of silencing complexes to these TEs during the establishment process. Similarly, human MORC2 is recruited by the HUSH complex to H3K9me3 marked heterochromatin and loss of MORC2 results in a decrease in H3K9me3 levels and transcriptional activation at a subset of HUSH loci^30^. Here again, MORC2 could act as a chromatin tether to promote the retention of the HUSH complex at its targets. Thus while MORCs in different organisms are clearly targeted to chromatin by different binding partners, the underlying mechanism of MORCs acting as tethers to assist silencing complexes may be conserved.

## Methods

### Plant materials and growth conditions

All plants in this study are grown at standard greenhouse conditions (22–25 ^◦^C, 16 h light/8 h dark). The following plant materials were used in this study: MORC7-FLAG in *morc7-1* (SALK_051729) background^25^, MORC4-FLAG in *morc4-1* background^25^, *morc hex* consisting of *morc1-2* (SAIL_893_B06) *morc2-1* (SALK_072774C) *morc4-1* (GK-249F08) *morc5-1* (SALK_049050C) *morc6-3* (GABI_599B06) and *morc7-1* (SALK_051729)^25^, *nrpd1-4* (SALK_083051), *suvh2 suvh9* (SALK_079574, SALK_048033), *nrpe1-11* (SALK_029919), *dms3-4* (SALK_125019C), *met1-3* (CS16394), *fwa-4*, *nrpe1-1*(EMS) retrogressed into *fwa-1*^40^.

### Plasmid construction

#### ZF-DMS3-3xHA

Modified pDT1H plasmid containing UBQ10 and C-terminal 3xHA^53^ was linearized by AatI digestion. ZF108 was fused to the N-terminus of DMS3 (cDNA) through overlapping PCR and subsequently ligated into linearized modified pDT1H with In-Fusion cloning system (Takara). Cloning primers are listed in Supplementary Table 1.

#### ZF-3xFLAG-MORC7

Entry vector containing cDNA of MORC7 was introduced into modified pMDC123 with pUBQ10::ZF108-3xFLAG through LR clonase (Invitrogen).

All plants in this study were grown under long day conditions (16 h light/8 h dark). Transgenic plants were generated by *Agrobacterium*-mediated transformation through floral dipping. T1 transgenic plants were selected with hygromycin B on ½ MS agar medium or sprayed with Basta in soil.

### ChIP-seq

Twelve ml of packed floral tissues were used for each ChIP^40^. For MORC7 ChIPs in RdDM mutants, the *pMORC7::MORC7-3xFLAG* transgene^25^ was crossed into *dms3-4* (SALK_125019C), *nrpe1-11* (SALK_029919), *nrpd1-4* (SALK_083051), *suvh2 suvh9* (SALK_079574 SALK_048033), and *nrpe1-11 nrpd1-4*. ChIP-seq was performed with either F2 or F3 plants homozygous null for the corresponding RdDM components, heterozygous or homozygous for the MORC7-FLAG transgene. All samples were crosslinked in vitro with Nuclei Isolation Buffer (50 mM HEPES, 1 M sucrose, 5 mM KCl, 5 mM MgCl_2_, 0.6% Triton X-100, 0.4 mM PMSF, 5 mM benzamidine, cOmplete EDTA-free Protease Inhibitor Cocktail (Roche)) supplemented with 1% formaldehyde for 12 min with rotation. Glycine was immediately added to stop the crosslinking. Lysate was filtered through Miracloth and centrifuged for 20 min at 2880 *g* at 4 ^◦^C. The pellet was resuspended in 1 ml of extraction buffer 2 (0.25 M sucrose, 10 mM Tris-HCl pH 8, 10 mM MgCl_2_, 1% Triton X-100, 5 mM BME, 0.1 mM PMSF, 5 mM Benzamidine, and 1x protease inhibitor cocktail tablet) and centrifuged at 12,000 *g* for 10 min at 4^◦^C. The pellet was resuspended in 500 µL extraction buffer 3 (1.7 M sucrose, 10 mM Tris-HCl pH 8, 2 mM MgCl_2_, 0.15% Triton X-100, 5 mM BME, 0.1 mM PMSF, 5 mM Benzamidine, 1x protease inhibitor cocktail tablet) and layered on top of 500 µL extraction buffer 3 (1.7 M sucrose, 10 mM Tris-HCl pH 8, 2 mM MgCl_2_, 0.15% Triton X-100, 5 mM BME, 0.1 mM PMSF, 5 mM Benzamidine, 1x protease inhibitor cocktail tablet) and centrifuged at 12,000 *g* for 1 h at 4 °C. The pellet was lysed with 400 µL Nuclei Lysis Buffer (50 mM Tris pH 8, 10 mM EDTA, 1% SDS, 0.1 mM PMSF, 5 mM Benzamidine, 1x protease inhibitor cocktail tablet). A total of 1.7 ml of ChIP Dilution Buffer (1.1% Triton X-100, 1.2 mM EDTA, 16.7 mM Tris pH 8, 167 mM NaCl, 0.1 mM PMSF, 5 mM Benzamidine, 1x protease inhibitor cocktail tablet) was added to the lysed nuclei. Chromatin was sheared with Bioruptor Plus (Diagenode) for 20 min with 30 s on and 30 s off and incubated with 7 ul of 1 mg/mL anti-FLAG M2 (Sigma F1804) antibody overnight at 4^◦^C. Chromatin-bound proteins were immunoprecipitated with Protein A and Protein G magnetic Dynabeads (Invitrogen) for 2 h at 4^◦^C. Dynabeads were washed twice with Low Salt Buffer (150 mM NaCl, 0.2% SDS, 0.5% Triton X-100, 2 mM EDTA, 20 mM Tris pH 8), once with High Salt Buffer (200 mM NaCl, 0.2% SDS, 0.5% Triton X-100, 2 mM EDTA, 20 mM Tris pH 8), once with LiCl Buffer (250 mM LiCl, 1% Igepal, 1% sodium deoxycholate, 1 mM EDTA, 10 mM Tris pH 8), and once with TE buffer (10 mM Tris pH 8, 1 mM EDTA). Elution was performed with 250 ul elution buffer (1% SDS, 10 mM EDTA, 0.1 M NaHCO_3_) by incubating at 65^◦^C with shaking twice. Foud hundred microliters of eluted complexes were reverse crosslinked by incubation at 65^◦^C overnight with the addition of 20 ul of 5 M NaCl followed by protease K treatment (20 ug in 10 mM EDTA and 40 mM Tris pH 8) at 45^◦^C for 1 h. DNA fragments were precipitated with EtOH overnight at −20^◦^C. Libraries were prepared with Ovation Ultra Low System V2 kits following the manufacturer’s instructions.

### Whole genome bisulfite sequencing

DNA from floral tissues were extracted with cetyl trimethylammonium bromide (CTAB)-based method. RNA was removed with PureLink RNase A (Invitrogen). A total of 300 ng of DNA was sheared to 200 bp with a Covaris S2 (Covaris). Libraries were prepared with the Epitect Bisulfite Conversion kit (QIAGEN) and the Ovation Ultralow Methyl-seq kit (NuGEN) following the manufacturer’s instructions.

### RNA-seq

Floral tissues from three-week-old seedlings were used for RNA extraction with Zymo Direct-zol RNA MiniPrep kit (Zymo Research) following manufacturer’s instructions. A total of 1 ug of total RNA was used for library preparation with TruSeq Stranded mRNA kit (Illumina). Libraries were sequenced on HiSeq 2500 or NovaSeq 6000 (Illumina).

### RT-qPCR

Leaf tissues from 3 to 4 week-old seedlings were extracted with Zymo Direct-zol RNA MiniPrep kit (Zymo Research). 400 ng–1 ug of total RNA was reverse transcribed into cDNA with Superscript III First Strand Synthesis Supermix (Invitrogen). qPCR was performed with iQ SYBR Green Supermix (Bio-Rad). Transcripts were normalized to the house keeping gene ISOPENTENYL PYROPHOSPHATE DIMETHYLALLYL PYROPHOSPHATE ISOMERASE 2 (IPP2). qPCR primers are listed in Supplementary Table 1.

### McrBC assay

Genomic DNA was prepared with CTAB method and treated with PureLink RNase (Invitrogen). A total of 100 ng of DNA was treated with McrBC (NEB) for 4 h at 37^◦^C. *FWA* promoter region was subsequently quantified by qPCR as described before. McrBC primers are listed in Supplementary Table 1.

### BS PCR

Rosette leaves from representative T2 and T3 were collected. DNA was extracted with a CTAB-based method followed by RNase A (Invitrogen) treatment to remove RNA. Bisulfite conversion was performed with Epitech Bisulfite Conversion Kit (Qiagen) following the manufacturer’s instructions. Three regions were amplified for the methylation analysis: chr4: 13038143-13038272, chr4: 13038356-13038499, and chr4: 13038568-13038695. The amplification was performed with Pfu Turbo Cx (Agilent). Libraries were made from purified PCR products using Kapa DNA hyper kit (Kapa Biosystems) with Illumina TruSeq DNA adapters. Libraries were sequenced on Illumina HiSeq 6000. BS PCR primers are listed in Supplementary Table 1.

### IP-MS and CLNIP-MS

Native IP-MS was performed with about 50 ml of packed floral tissues^25^. Floral tissues were ground into fine powder using RETCH homogenizer and resuspended in 25 ml of IP buffer (50 mM Tris-HCl pH 8.0, 150 mM NaCl, 5 mM EDTA, 10% glycerol, 0.1% Tergitol, 0.5 mM DTT, and cOmplete EDTA-free Protease Inhibitor Cocktail (Roche)). Clumps were broken by Dounce homogenizer. Lysate was filtered through Mirachoth and centrifuged at 20,000 *g* for 10 min at 4 ^◦^C. Supernatant were incubated with 200 ul of anti-FLAG M2 magnetic beads (Sigma) at 4 ^◦^C for 2 h with rotation. Beads were washed 5 times with IP buffer. Bead bound proteins were eluted with 300 ul of 250 ug/ml 3xFLAG peptide (Sigma), with vigorous mixing at 37 ^◦^C for 15 min each elution for a total of two elutions. The eluted proteins were subjected to trichloroacetic acid precipitation and mass spectrometric analysis.

Crosslinked IP was performed with 15 g of floral tissues and resuspended in Nuclei Isolation Buffer (50 mM HEPES, 1 M sucrose, 5 mM KCl, 5 mM MgCl_2_, 0.6% Triton X-100, 0.4 mM PMSF, 5 mM benzamidine, cOmplete EDTA-free Protease Inhibitor Cocktail (Roche)) to reach a final volume of 40 ml and supplemented with 1% formaldehyde for 12 min with rotation. Glycine was added immediately to stop the crosslinking. Clumps were broken by Dounce homogenizer and lysate was filtered through Miracloth and centrifuged at 1500 *g* for 10 min at 4 ^◦^C. Nuclei pellet was resuspended and washed with NRBT buffer (20 mM Tris-HCl pH 7.5, 2.5 mM MgCl_2_, 25% glycerol, 0.2% Triton X-100) twice and resuspended in 6 ml of RIPA buffer (1x PBS, 1% NP-40, 0.5% sodium deoxycholate, 0.1% SDS). Resuspended nuclei were split into 3x 2 ml aliquots for sonication for 20 min (30 s on/30 s off) with Bioruptor Plus (Diagenode). Sheared lysate was centrifuged at 8000 *g* for 15 min at 4^◦^C and combined supernatant was incubated with 200 ul of FLAG-M2 magnetic beads (50% slurry, Sigma) for 2 h at 4 ^◦^C with rotation. Beads were washed, eluted, and precipitated as described in native IP.

The TCA precipitated samples were resuspended in 50 μl of digestion buffer (8 M urea, 100 mM Tris pH 8.5). Each sample was reduced and alkylated by adding TCEP and iodoacetamide to final concentrations of 5 mM and 10 mM, incubated at room temperature in the dark for 20 min, and then digested by 0.1 μg of Lys-C (Thermo Scientific, 90051) and 0.8 μg Trypsin (Thermo Scientific, 90057) proteases at 37 °C overnight. The digested samples were quenched by the addition of formic acid to 5% (v./v.) final concentration. Finally, each sample was desalted via C18 tips (Thermo Scientific, 87784) and reconstituted in 15 μL of 5% formic acid before analyzed by LC–MS/MS.

Digested peptides were resuspended in 5% formic acid and fractionated online using a 25 cm long, 75 uM inner diameter fused silica capillary packed in-house with bulk C18 reversed phase resin (length, 25 cm; inner diameter, 75 uM; particle size, 1.9 μm; pore size, 100 Å; Dr. Maisch GmbH). The 140-minute water–acetonitrile gradient was delivered using a Dionex Ultimate 3000 UHPLC system (Thermo Fisher Scientific) at a flow rate of 300 nl/min (Buffer A: water with 3% DMSO and 0.1% formic acid and Buffer B: acetonitrile with 3% DMSO and 0.1% formic acid). Fractionated peptides were ionized and analyzed by tandem mass spectrometry (MS/MS) Orbitrap Fusion Lumos mass spectrometer (Thermo Fisher Scientific). Data was acquired using a Data-Dependent Acquisition (DDA) method comprised of a full MS1 scan (Resolution = 120,000) followed by sequential MS2 scans (Resolution = 15,000) to utilize the remainder of the 3 second cycle time.

Data analysis including peptide and protein identification was performed using MS2 spectra were searched using the ProLuCID algorithm against Arabidopsis reference proteome followed by filtering of peptide-to-spectrum matches (PSMs) by DTASelect using a decoy database-estimated false discovery rate of <1%.

### Bioinformatic analysis

#### ChIP-seq

All libraries were sequenced at a length of 50 bps with HiSeq 2500 or NovaSeq 6000 platforms following manufacturer’s instructions (Illumina). Raw reads were aligned to *the Arabidopsis* reference genome (TAIR10) with Bowtie2 (v2.1.0)^54^, allowing only uniquely mapped reads with perfect matches. Duplicated reads were removed with Samtools (v1.9)^55^. Peaks were called using MACS2 (v2.1.1)^56^. To increase sequencing depth, three independent ChIPs of MORC7 in wild-type and two independent MORC7 ChIPs in RdDM mutants were pooled for peak calling.

#### Whole Genome Bisulfite Sequencing (BS-seq) and analysis

BS-seq reads were mapped to TAIR10 reference genome by bsmap (v2.90) with allowing 2 mismatches and 1 best hit (-v 2 -w 1)^57^. Reads with three or more consecutively methylated CHH sites were considered as non-converted reads and removed from the analyses. DNA methylation levels were calculated by #C/ (#C + #T). Differential Methylated Regions (DMRs) were called by methdiff function with every 100 bp bin for where the difference in CG, CHG, and CHH methylation is at least 0.4, 0.2, and 0.1, respectively.

#### *met1* RNA seq analysis

met1 RNA seq data were downloaded from NCBI Gene Expression Omnibus (GEO) as accession GSE93584^58^. Cleaned short reads were aligned to reference genome TAIR10 by Bowtie2 (v2.1.0)^54^, and expression abundance was calculated by RSEM (v1.3.1) with default parameters^59^.

#### Bisulfite PCR analysis

Only reads with sequences matching primers used for bisulfite PCR were used for analysis. Primer sequences were trimmed off reads and reads were aligned to a ‘genome’ consisting of the target amplicons (both wild-type and transgene sequences with polymorphisms) using bismark^60^. Fully unmethylated reads were extracted and quantified for each amplicon using a custom script (available upon request).

### Reporting summary

Further information on research design is available in the Nature Research Reporting Summary linked to this article.

## Supplementary information

Supplementary Information

Reporting Summary

## Data Availability

Data supporting the findings of this work are available within the paper and its Supplementary Information files. The datasets generated and analyzed during the current study are available from the corresponding author upon request. All high-throughput sequencing data generated in this study is accessible at NCBI’s Gene Expression Omnibus (GEO) via GEO Series accession number GSE160285. The mass spectrometry proteomics data have been deposited to the ProteomeXchange Consortium via the PRIDE partner repository with the dataset identifier PXD026674. Source data underlying Figs. 5 and 6 are provided as a Source Data file. Souse data are provided with this paper. Source data are provided with this paper.

## Source data

Source data file

## Code availability

All custom scripts are available from the corresponding author upon request.
