# Supplementary material for: Arabidopsis MORC proteins function in the efficient establishment of RNA directed DNA methylation
Source: Nat Commun. 2021 Jul 13;12:4292. doi: 10.1038/s41467-021-24553-3 (PMC8277788; doi:10.1038/s41467-021-24553-3)
Supplement: Supplementary file 1 — Supplementary Information [file 41467_2021_24553_MOESM1_ESM.pdf]

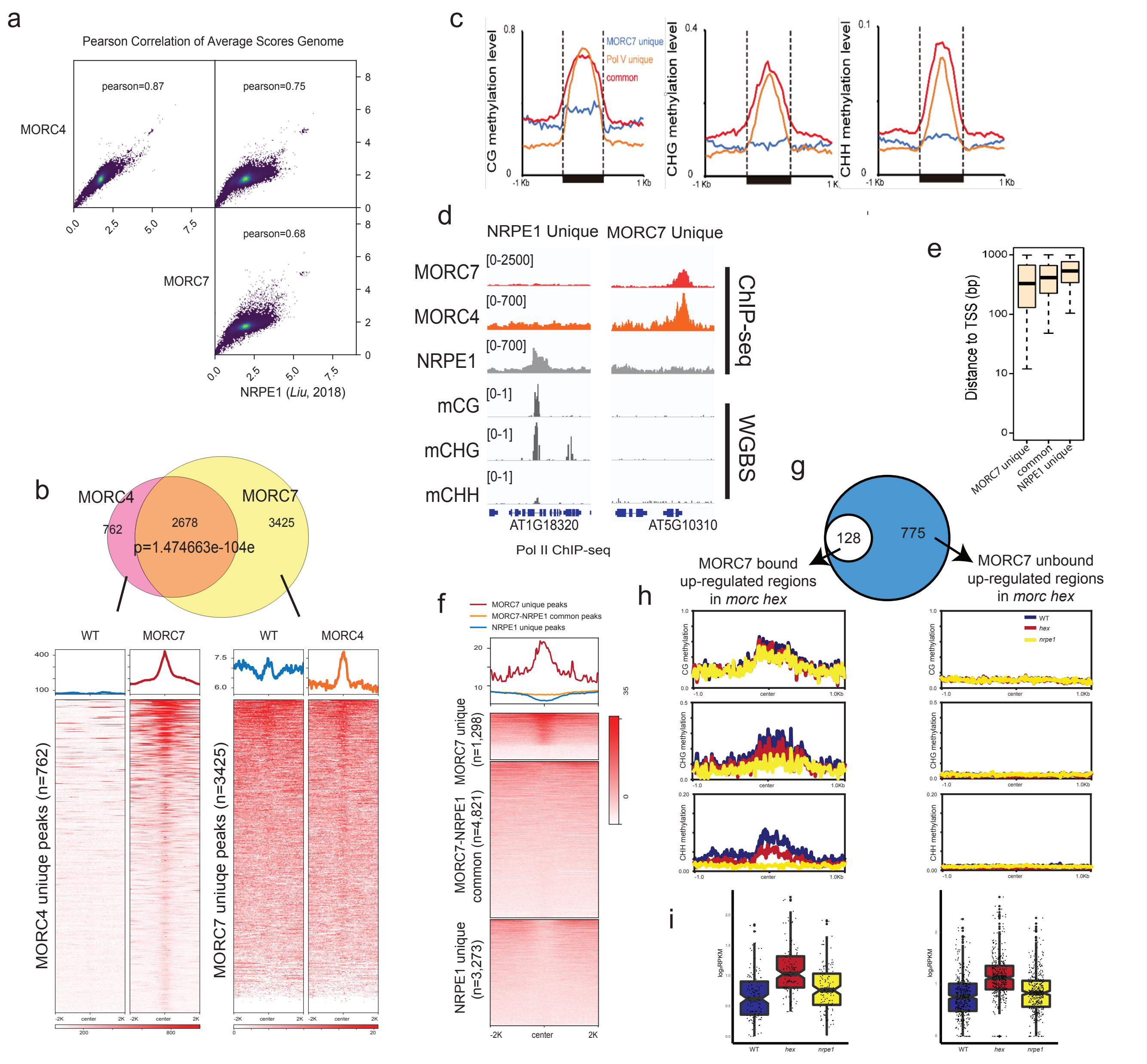

Supplementary Fig. 1. MORC4 and MORC7 chromatin localization, related to Figure 1. a Pearson correlation of MORC4-FLAG, MORC7-FLAG and NRPE1 ChIP-seq. **b** Overlap between MORC4 and MORC7 binding sites (top) (p = 1.474663e-104e, Hypergeometric test), enrichment of MORC4 and MORC7 ChIP signals (RPKM sample/control) over MORC7 and MORC4 specific regions respectively (bottom). **c** Enrichment of CG (left panel), CHG (middle panel) and CHH (right panel) DNA methylation over MORC7 unique (blue), MORC7-NRPE1 common (red) and NRPE1 unique (orange) peaks. **d** Screenshot of a representative locus of NRPE1 unique (left) and MORC7 unique peak (right). **e** Boxplot showing the distance of MORC7 unique, MORC7-NRPE1 common and NRPE1 unique peaks to the transcription start site (TSS) of the nearest gene. **f** Profile and heatmap of Pol II ChIP signals (RPKM sample/control) over MORC7 unique, MORC7-NRPE1 common and NRPE1 unique peaks. **g** Venn diagram showing regions that are upregulated in *hex*. Regions that have MORC7 bound within 1Kb (MORC7 bound) are in light blue (n=128) and regions that are MORC7 un-bound are in dark blue (n=775). **h** DNA methylation over MORC7 bound regions (left) and MORC7 unbound regions (right). **i** Expression (log_2RPKM) over the MORC7 bound (left) and MORC7 unbound (right) regions in WT (blue), hex (red) and nrpe1 (yellow).

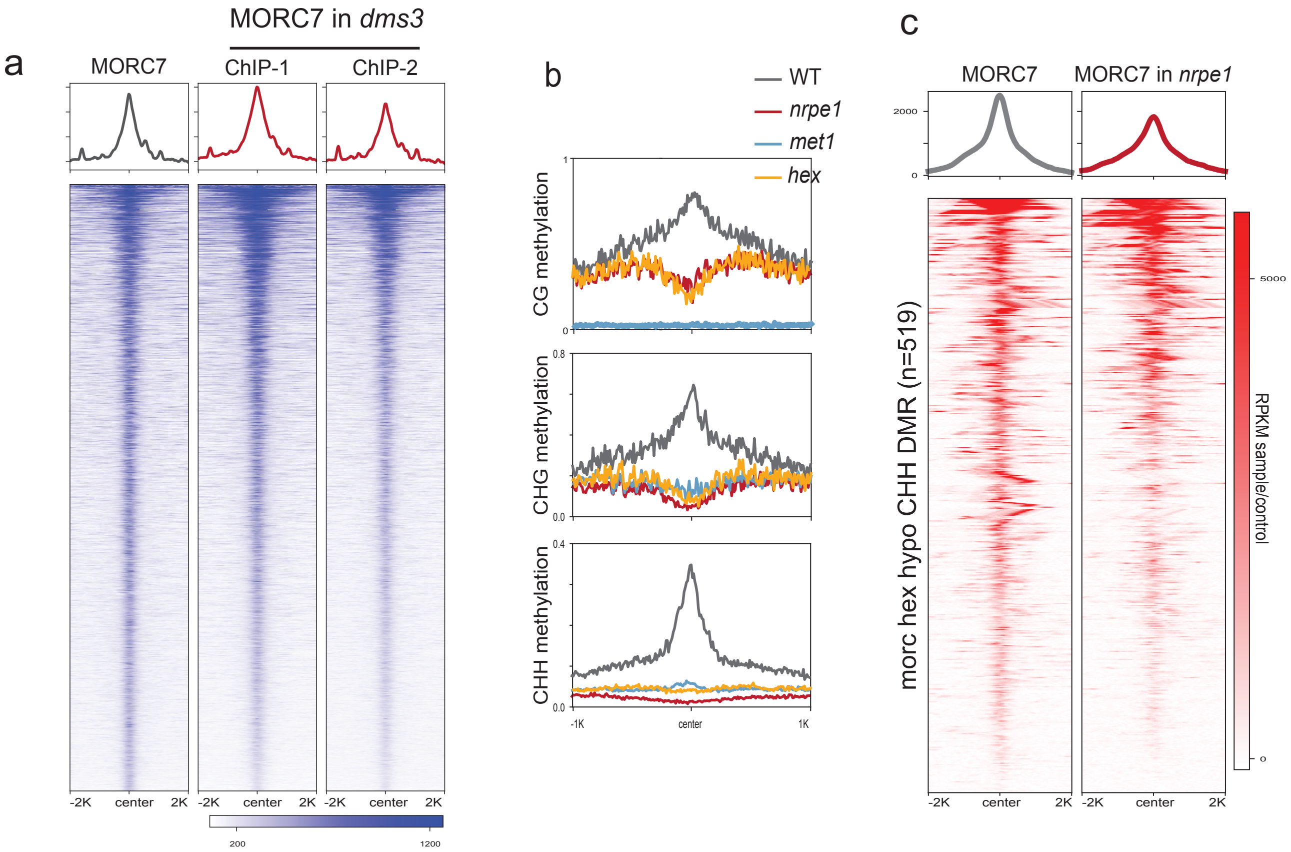

Supplementary Fig. 2. MORC7 binding in *dms3-4* and over *morc hex* hypo-CHH DMRs (fragile sites), related to Figure 2. a Enrichment of MORC7 ChIP signals (biological replicate of Fig. 2d) (RPKM sample/control) in *dms3* (right) and its corresponding WT control (left). **b** Methylation level over the *hex* hypo-CHH DMRs in WT (grey), *hex* (orange), *nrpe1* (red) and *met1* (blue) (n = 519). **c** Enrichment of MORC7 ChIP signal (RPKM sample/control) over the same *hex* hypo-CHH DMR in **b.**

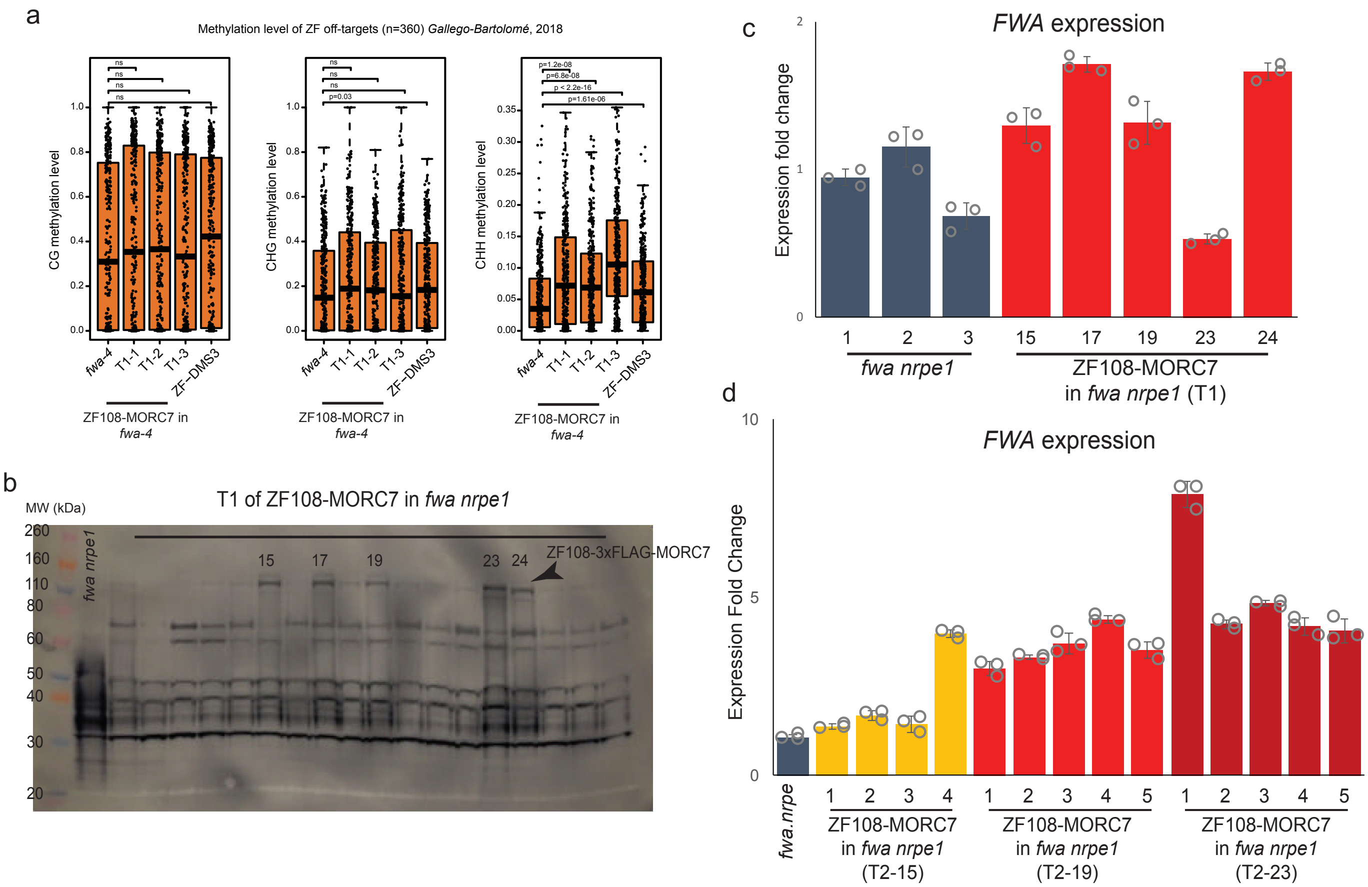

Supplementary Fig. 3. MORC7 mediated repression on *FWA* is dependent on RdDM, related to Figure 4. a Boxplot showing 360 ZF ectopic sites that gained DNA methylation in the three independent T1 plants showed in Fig 5a. Wilcoxon test was used to determine significance. (ns: p value > 0.05). **b** anti-FLAG western blot showing the expression of ZF108-3xFLAG-MORC7 in the T1 plants displayed in **a.** Arrow indicates the band with expected size. T1 lines with their corresponding numbers were chosen for further analysis. **c** qRT-PCR analysis showing *FWA* expression level relative to *fwa npre1* in five independent T1 plants. Error bars represent the mean ± s.d. of 3 technical replicates. **d** qRT-PCR analysis showing *FWA* expression level relative to *fwa nrpe1* in the T2 progenies of three T1 plants. Error bars represent the mean ± s.d. of 3 technical replicates.

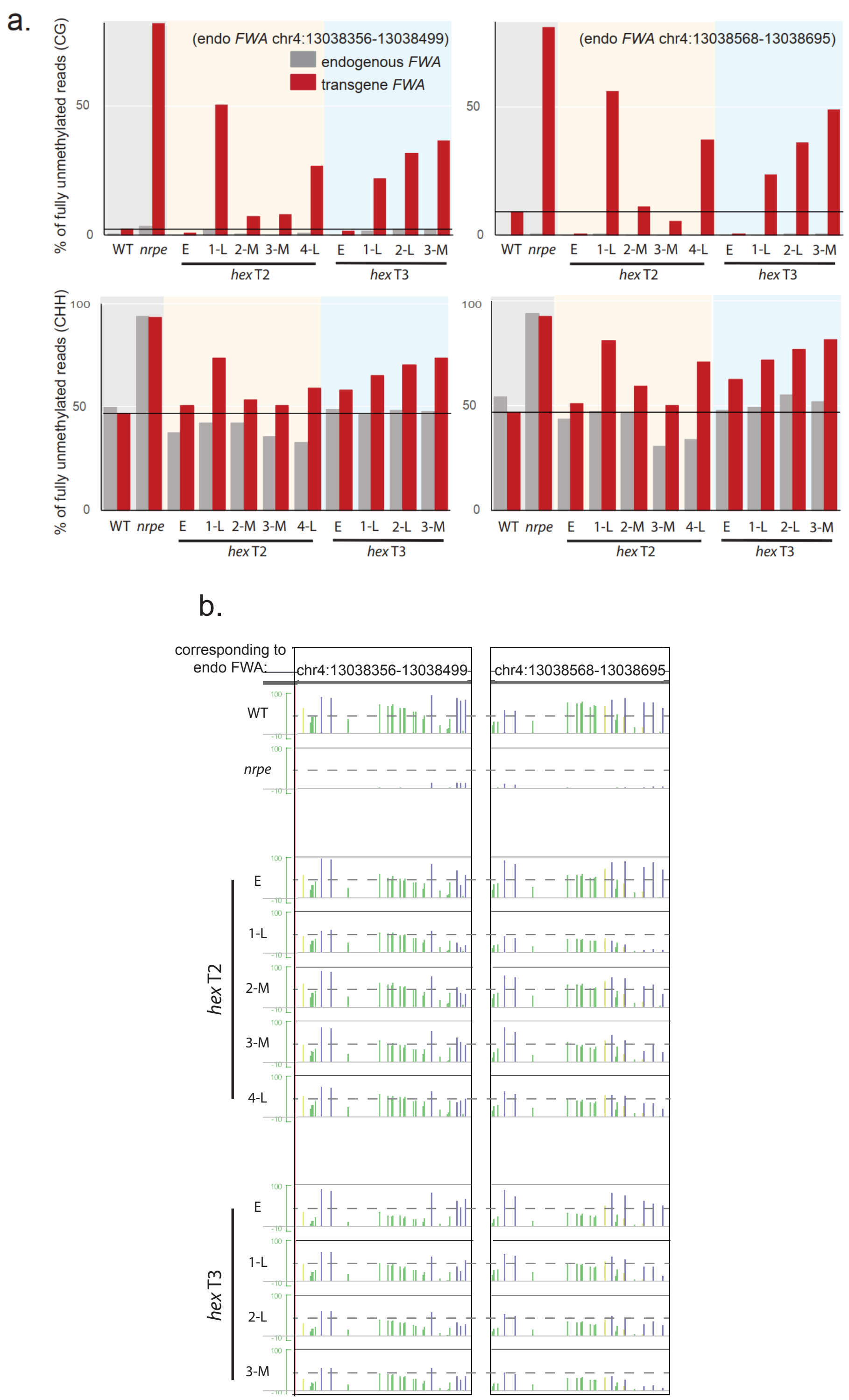

Supplementary Fig. 4. Establishment of DNA methylation is attenuated over *FWA* transgene.
a Percentage of fully CG unmethylated reads (upper panels) or fully CHH unmethylated reads (lower panels) from the endogenous *FWA* (grey) and *FWA* transgene (red) over two regions within the promoter. The dark lines indicate the levels of fully unmethylated *FWA* transgene in WT. E: early flowering; M: Intermediate flowering; L: late flowering. **b** Methylation tracks of the *FWA* transgene over the same regions as in a. CG:blue; CHG, yellow; CHH, green.

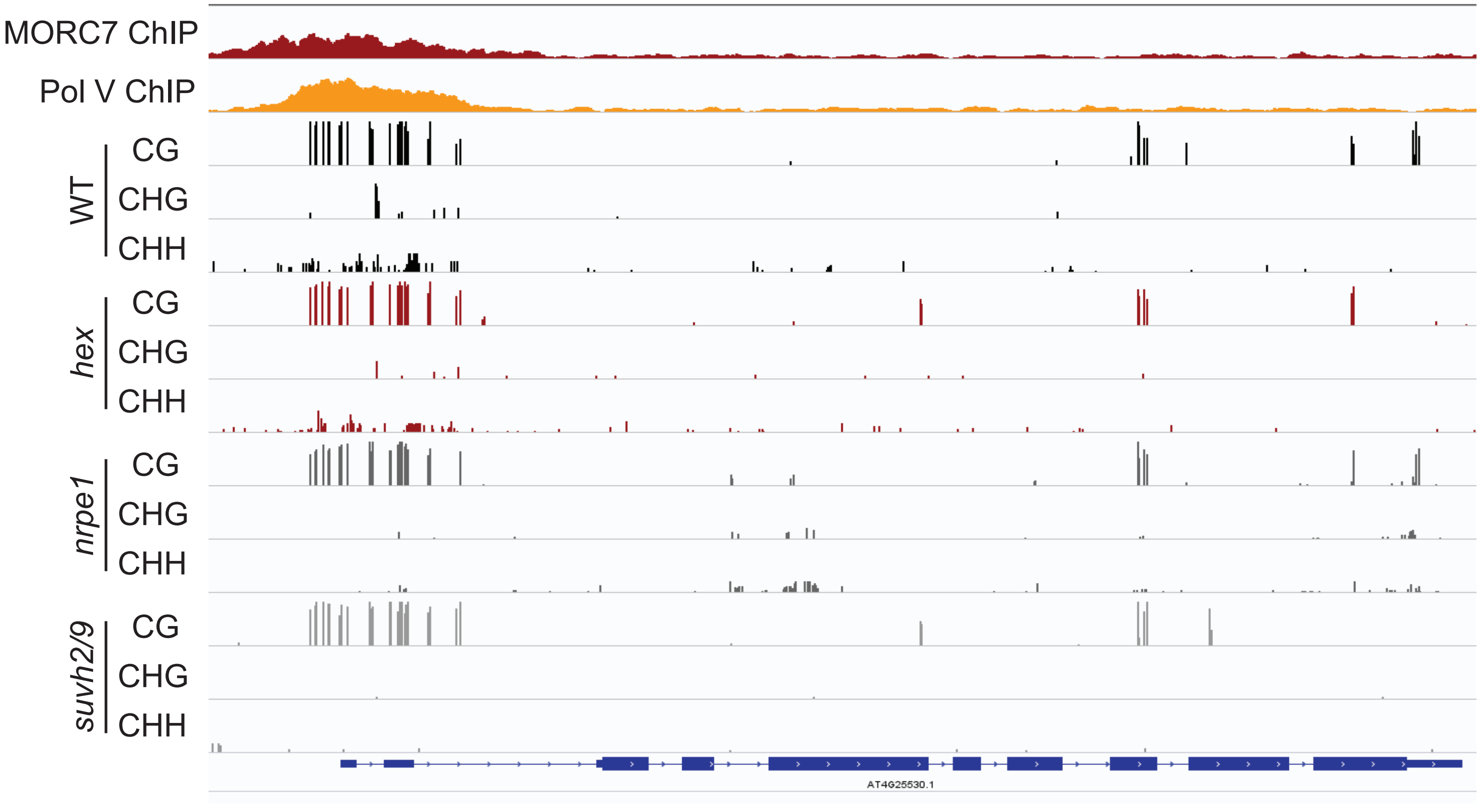

Supplementary Fig. 5. Methylation over endogenous *FWA* promoter is not affected in *hex*, related to Figure 5. Screenshot of MORC7 ChIP seq (crimson), Pol V ChIP (orange) over *FWA* locus (AT4G25530) as well as DNA methylation level in WT (black), *hex* (crimson), *nrpe1* (dark grey) and *suvh2/9* (light grey) in CG, CHG and CHH context.

Supplementary Table 1: Primers used in this study

Genotyping primers:

morc1-2 SAIL_893_B06	TTGCAGTTTGGAACCAAAATC	TGAGTTTTGACGACGATGATG
morc2-1 SALK_072774 C	CTACTCAGAGCGTTGGCATTC	GTTGTAGCTGTATGGGGCTTG
morc4-1 GK-249F08	TCAGGAAAGATTTCACGAATTG	ACCTGCAGAAACTTCCCAATC
morc5-1 SALK_049050 C	GTTGGGATAGATAAGGCGACC	TGTCGAGAAATCGTTCCTTTG
morc6-3 GABI_599B06	ACATCTTCCAATGGCTGAATC	GCTGGTGTCACTTCTTCATCC
morc7-1 SALK_051729	GTCGAAAGGATGTGAGAAACG	TTCCATTCAATTGCTTGGTTC
nrpe1-11 SALK_029919	ATTTCTTCTTTGATGGGGGAG	TGTCGTGGATATGACCATTTG
nrpd1-4 SALK_083051	TGGGTTTGCCATTTTCATATC	GCATGCTTGAGTAAAAGGTGC
suvh2 SALK_079574	GCCCAGGTGTTGTTTCTGTC	CCATATCGCTCCTCCGTCTA
suvh9 SALK_048033	CACACTAGTAGCTATTGGCTCCCCATTA	TCTCTGATAAACTCGTCAAACCCATTTCG
dms3-4/idn1-1	CTGCCTTGAAAACCTGAGGTAC	TCTGATAAGATGCATTCCATGG
met1-3 CS16394	GATTGTGTCTCTACTACAGAGGC	GTTAAGCTCATTCATAGCCTTGC
nrpe1-1	CGTGGCATCATGAAACATTCACTGC	CCATCTGATGCTCTATCTTTGATTGAGATGT

BS PCR:

FWA region 1 chr4:13038356-13038499	CTCATATATACCTTATCCCATTCAACATTCATA	AAGATYTGATATTTGGYTGGAAAAAAYAATAATAAT
FWA region 2 chr4:13038568-13038695	CRCTCTTTATCCCATTCAACATTCATAC	TTTGGTTGAAAAAAATAATAAAAATTTGATTGTYAGTAT

Cloning primers:

Primer	Description	Sequence
JP22356	N terminal ZF infusion into pDT Forward	AAAGACCGGTCAATTGGACGTCATGGTTGGGTACCAACAAATGG
JP22357	ZF-linker-DMS3 (overlap Reverse)	GATACATCCCACCAGAGCCTCCGAGTCCGGTGTGAGTCCTTTG
JP22358	ZF-linker-MORC7 (overlap Forward)	AAAGGACTCACACCGGACTCGGAGGCTCTGGTGGGATGGACAACAGTATTCACGT
JP22359	DMS3 infupDT infusion into pDT Reverse (no stop codon)	GAACGATTTAATTAATTAGACGTCTCATCTGGGTGTGTTCATTGG

qPCR primers:

Description	Forward primer	Reverse primer
McrBC over *FWA* promoter	TTGGGTTTAGTGTTTACTTG	GAATGTTGAATGGGATAAGGTA
FWA expression	TTAGATCCAAAGGAGTATCAAAG	CTTTGGTACCAGCGGAGA
IPP2 expression	GTATGAGTTGCTTCTCCAGCAAAG	GAGGATGGCTGCAACAAGTGT
